# CARDIAC ABNORMALITIES INDUCED BY BENZENE EXPOSURE FROM THE FLARING DISASTER AT THE BP REFINERY PLANT IN TEXAS CITY

**DOI:** 10.13075/ijomeh.1896.02480

**Published:** 2025

**Authors:** Mark A. D'andrea, Nadish Garg, Shubham Trehan, G. Kesava Reddy

**Affiliations:** 1 University Cancer and Diagnostic Centers, Houston, TX, USA; 2 Dayanand Medical College and Hospital Ludhiana, Punjab, India

**Keywords:** pulmonary function, cardiac function, benzene poisoning, urinary phenol, flaring disaster, toxic emission

## Abstract

**Objectives:**

Benzene exposure is associated with multiple adverse health effects on the human's biological systems including its cardiac, pulmonary, respiratory, renal, liver, and other organs' function. The purpose of this study is to assess the adverse health effects of benzene exposure on the cardiac functions in subjects affected by a flaring incident at the British Petroleum (BP) refinery in Texas City, Texas, USA.

**Material and Methods:**

A total of 1790 evaluable subjects who were exposed to benzene were included in the study. Using the patients' medical charts, the authors analyzed the data on various heart rate parameters as well as on the pulmonary function, the serum levels of β_2_-microglobulin, and the amount of urinary excretion of phenol.

**Results:**

Of the 1790 subjects, 1083 experienced some type of cardiac function abnormality as assed by electrocardiogram (EKG) testing following their exposure to benzene. Normal cardiac function was preserved in 707 subjects despite their exposure to benzene. Regardless of the changes in their overall cardiac function, most benzene exposed subjects experienced some changes in various heart rate parameters such as P wave duration, PR interval, PR segment, QRS duration, QT interval, QTC interval, P wave axis, QRS axis, and T wave axis. Similarly, alterations in their pulmonary function test (PFT), β_2_-microglobulin levels, and urinary excretion of phenol were observed in benzene exposed subjects regardless of the changes in cardiac function. Furthermore, the incidence of the abnormality of various heart rate parameters was found to be 2–10 fold higher in the benzene exposed subjects compared with the general population.

**Conclusions:**

Environmental benzene exposure from the BP flaring incident pose significant health risks including specific alteration in cardiac and pulmonary functions in those subjects exposed to benzene.

## INTRODUCTION

Benzene is a volatile organic toxic chemical produced in large quantities by the petroleum refining industries [1]. Benzene ranks in the 20 of the most abundantly produced chemicals in the United States [2]. Commercially, numerous industries utilize benzene as an important intermediate to produce many chemicals including resins, synthetic fibers, and various polymers. Petrochemical and petroleum refining industries are the major source of benzene production, and its emissions occur most commonly during the refining processes [3]. The emission of benzene into the air by these chemical industries accounts >3.04 million kg annually [4]. Therefore, benzene is considered as one of the main contributors to air pollution in the environment. Benzene exposure is associated with significant health effects in humans [5–8]. Notably, the toxic effects of benzene exposure on the hematopoietic system are important due to the risk of developing various cancers including leukemia, and lymphoma, as well as other solid malignancies [7,9–11]. In addition, benzene exposure is associated with a wide range of adverse effects on the function of the hematological, hepatic, respiratory, cardiovascular, nervous, immune, renal, and reproductive systems [12–15]. Thus, communities that are in close proximity to the petroleum refineries have a heightened risk of exposure to benzene.

It is well established that benzene exposure has significant acute and chronic effects on human health. Both short-and long-terms effects of benzene can impair normal functions of multiple organs, particularly the hematopoietic, digestive, nervous, pulmonary, cardiovascular, renal, and immune systems [16–18]. The acute exposure to benzene may cause narcosis, headache, dizziness, drowsiness, confusion, respiratory irritation, tremors, visual abnormalities and loss of consciousness [17]. In addition, acute benzene toxicity symptoms include depression of the central nervous system, cardiac arrhythmias, and, if exposure levels are high enough, shortness of breath and respiratory failure. Whereas hematotoxicity, neurotoxicity, immunotoxicity, and carcinogenicity are the most significant health impacts of short-term and long-term benzene exposure. Additionally, benzene exposure can cause aplastic anemia, chromosomal abnormalities, and carcinogenesis in the bone marrow. Moreover, chronic benzene exposure is linked to increased inflammation and oxidative stress, contributing to DNA damage and the development of various malignancies including acute myeloid leukemia, myelodysplastic syndrome and other types of cancers [19,20].

Although the mechanism of benzene-induced multi organ toxicity is not completely understood, benzene metabolites such as phenol, benzoquinone, muconaldehydes, hydroquinone, and catechol play an integral role in its toxicity [21]. Studies have shown that these benzene metabolites are directly involved in its cytotoxic and genotoxic effects [22,23]. More specifically, when benzene is metabolized into various metabolites by the hepatic enzyme CYP2E1, reactive oxygen species such as superoxide anions (O_2_), hydroxyl radicals (OH) and hydrogen peroxide (H_2_O_2_) are generated [24]. It is believed that this oxidative stress contributes to the immune systems dysfunction and reduced immune surveillance leading to the inflammatory and cytotoxic effects of benzene on multiple tissues [25]. Because of its high reactivity to form adducts with proteins and DNA in the bone marrow, benzoquinone is known to induce myelotoxicity. Importantly, the resultant adducts interfere with cellular functions and cause damage to hematopoietic cells. In addition, those adducts can cause chromosomal aberration, oxidative stress, gene expression alteration, error-prone DNA repair, epigenetic regulation, apoptosis, and disruption of tumor surveillance.

A 2010 flaring disaster occurred at the British Petroleum (BP) refinery facility that led to the release of a massive amount of toxic chemicals into the skies of Texas City, Texas, USA. This disaster lasted for 40 days and led to the release of >227.3 thousand kg of toxic chemicals, including >7.7 thousand kg of benzene into the surrounding communities [26,27]. This flaring incident contaminated the air with toxic emissions and threatened the health of the residents of Texas City and the surrounding communities. The Galveston County District Clerk's Office estimates that >50 000 residents were affected by the BP refinery disaster. Although the hemotoxic effects of benzene exposure have been studied extensively, little is known about the toxic effects of benzene exposure on myocardial functions. Previously, the authors have reported that benzene exposure has led to significant alterations in the hematological and hepatic functions among the people in the affected communities of the BP refinery disaster [28–30]. To further substantiate these findings, the authors conducted a large study to evaluate the effect of benzene exposure on the cardiac functions of the residents affected by the BP refinery flaring disaster.

## MATERIAL AND METHODS

### Subjects

**Figure 1.**
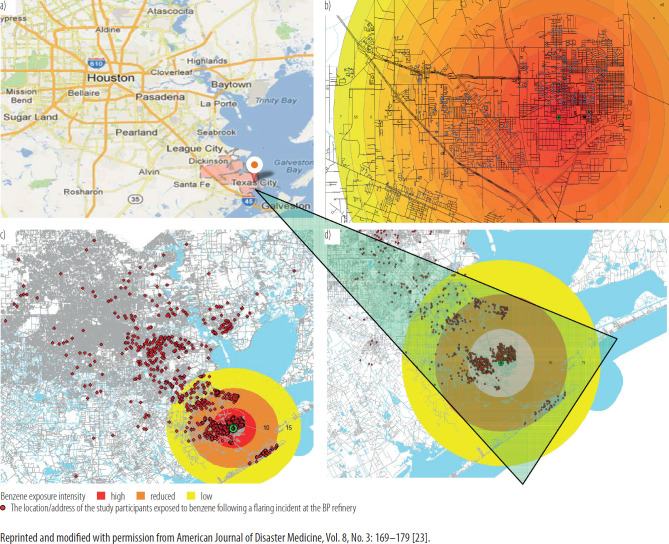
The location of the 2010 disaster of British Petroleum (BP) refinery facility in the northern parts of the Texas City, Texas, USA: a) location of Texas City, b) intensity of benzene exposure from BP incident surrounding neighborhoods of Texas City, c) the location/address of the study participants exposed to benzene following a flaring incident at the BP refinery and surrounding areas, d) a closer look at the affected area

This study was approved by an Institutional Review Board. The methodology for subject selection and medical evaluation was reported previously [28–30]. Briefly, the authors reviewed the medical charts of subjects who underwent clinical evaluations in June 2010 – October 2012. Residential areas affected by the BP refinery emission were identified and the residents exposed to the emission were included in the study (Figure 1). The subjects self-reported exposure to benzene following the flaring disaster. Specifically, these subjects experienced a prolonged and involuntary exposure to benzene for up to 40 days following the disaster that occurred on April 6, 2010 and lasted through May 16, 2010. The study was conducted according to the ethical principles of the Declaration of Helsinki. To comply with the Health Insurance Portability and Accountability Act (HIPAA), confidentiality of their information was secured by utilizing text encryption, password protection and limited personnel involvement.

### Chart review and data gathering

Study investigators reviewed the medical records to evaluate the clinical data of the subjects who were exposed to benzene. Medical examination of the benzene exposed subjects was carried out after the 40 days of the flaring disaster event and took approx. 19 months to complete all clinical procedures.

### Evaluation of electrocardiogram parameters

Clinical data on electrocardiogram (EKG) parameters including P wave duration, PR interval, PR segment, QRS duration, QT interval, QTC interval, P wave axis, QRS axis, and T wave axis were evaluated. Deviations from the normal range values (Table 1) of various EKG parameters were noted as an abnormality in the cardiac function in the benzene exposed subjects. In addition, based on the intensity of the changes in the cardiac function, the authors then categorized the cardiac abnormalities as normal, mild, moderate, or severe as shown in Table 2.

**Table 1.** Heart rate parameters – normal ranges

Parameter	Normal range
P wave duration	50–120 ms
QRS duration	60–100 ms
QT interval	<400 ms
QTC interval	<400 ms
P wave axis	0° to +75°
QRS wave axis	+30° to +190°
T wave axis	0° to 90°

**Table 2.** Criteria for categorizing the cardiac function abnormalities in benzene exposed subjects who underwent clinical evaluations in June 2010 – October 2012

Cardiac function	Criteria
Normal	– normal sinus rhythm – normal EKG with no abnormalities – sinus rhythm within normal limits – sinus rhythm with sinus arrhythmia normal
Mild	– sinus bradycardia – sinus arrhythmia/irregular sinus bradycardia – low voltage EKG – right axis deviation – regular atrial pacing – low voltage QRS with pulmonary disease – sinus bradycardia low QRS voltage in chest leads – sinus bradycardia marked left axis deviation – sinus bradycardia minor intraventricular conduction defect – sinus bradycardia moderate voltage criteria for LVH – sinus bradycardia nonspecific T wave abnormalities – sinus bradycardia RSR in V1/V2 consistent with right ventricular delay – sinus bradycardia minor IV conduction defect suggesting pericarditis – sinus tachycardia with low QRS voltage and nonspecific ST/T wave changes – sinus tachycardia with frequent premature ventricular contractions – supraventricular extrasystoles – undetermined rhythm with nonspecific intraventricular conduction block
Moderate	– incomplete RBBB/LBBB – non-specific T wave changes – first degree heart block – sinus rhythm, minor conduction delay – bilateral ectopic atrial rhythm – early repolarization – premature atrial contraction – juvenile T waves – minor IV conduction delay/abnormal ventricular pathway – occasional premature ventricular contractions – short PR interval – sinus rhythm – supraventricular extrasystoles – non-specific intraventricular conduction block
Severe	– complete RBBB/LBBB – atrial fibrillation, atrial flutter – possible left atrium enlargement – myocardial ischemia – pericarditis – LVH/RVH – ST changes in anterior/septal/lateral leads – QT interval prolongation – old infarct – bilateral abnormal irregular supraventricular rhythm – ectopic atrial rhythm – fascicular block – marked bradycardia (<50 bpm) – RSR in V1/V2 consistent with ventricular delay – atrioventricular dissociation – sinus tachycardia with old infarct low QRS voltage

EKG – electrocardiogram; LBBB – left bundle branch block; LVH – left ventricular hypertrophy; RBBB – right bundle branch block; RSR – R wave, S wave, R wave (pattern on EKG); RVH – right ventricular hypertrophy; ST – ST segment (portion of the EKG wave).

### Pulmonary function testing

Concurrently, pulmonary function tests (PFTs) were performed using an electronic spirometer. The precise technique used in the measurement of the various lung function tests for the present study were based on the operation manual of the instrument with special reference to the official recommendations of the European Respiratory Society/American Thoracic Society of Standardization of Spirometry [31]. The subjects were given standard instructions about the procedure. The spirometric tests were performed in the sitting position and each subject had a nose clip applied during the maneuver. Based on the intensity of changes, the abnormality in the pulmonary function has been categorized into normal, mild, moderate, and severe groups.

### Quantification of β_2_-microglobulin

Laboratory tests on blood samples of the benzene exposed subjects were performed by an accredited laboratory facility (LabCorp, Laboratory Corporation of America, Houston, TX, USA). The amount of β_2_-microglobulin in the serum of benzene exposed subjects was quantified using a standard enzyme-linked immunosorbent assay. The serum levels of β_2_-microglobulin <2.0 µg was regarded as normal and ≥2.0 µg was regarded as abnormal.

### Analysis of urinary phenol

Laboratory tests on urine samples of the benzene exposed subjects were performed by an accredited laboratory facility (LabCorp). Urinary phenol was assessed as a benzene metabolite using Agilent 5980 GC system (Agilent Technologies, Wilmington, DE, USA) in the benzene exposed subjects. All analyses were conducted by LabCorp unaware of the subjects' exposure status. Depending on the amount of excretion of urinary phenol, the subjects were categorized into normal, mild, moderate, and severe groups.

### Statistical analysis

Descriptive statistics were used to evaluate data variables, producing means and standard deviations for the intensity of EKG changes. To quantify the intensity of the changes in the cardiac function, the authors computed the total score derived from categories of mild being representing 1, moderate representing 2, and severe representing 3 as shown in Table 2.

Student's t-test was performed to determine significant differences among various indices. The significance level was predetermined at an α level of 0.05. The prevalence of benzene exposure induced changes was expressed as a percentage either a positive or negative in relation to the corresponding variables.

## RESULTS

A total of 2350 subjects who were affected by the BP flaring disaster were screened. Electrocardiogram data was not available for 560 of the 2350 subjects and therefore they were excluded from the study. The remaining 1790 subjects who had EKG data were included in the study. The results presented in Table 3 show the demographics of the subjects who were exposed to benzene. Of the 1790 subjects who were included in the study, there were 58% (N = 1044) male and 42% (N = 746) female subjects. Among 1790 subjects, 95% (N = 1695) were adults ≥18 years, and 5% (N = 95) were children <18 years. Tobacco smokers accounted for 28% (N = 506) and nonsmokers accounted for 72% (N = 1284). The median time from the flaring disaster event to time of medical evaluation and testing was 142 (range 72–564) days (Table 3).

**Table 3.** Demographics of the benzene exposed subjects who underwent clinical evaluations in June 2010 – October 2012

Variable	Participants (N = 1790)
Subjects with cardiac abnormalities [n (%)]	1083 (61)
Age of all subjects [years] (M (range))	41 (7–89)
Gender [n (%)]	
male	1044 (58)
female	746 (42)
Children (<18 years) (N = 95, 5%)	
age [years] (M (range))	12.5 (7–18)
Adults (≥18 years) (N = 1695, 95%)	
age [years] (M (range))	54 (19–89)
Time from disaster event to laboratory testing [days] (Me (range))	142 (72–564)
Smoking [n (%)]	
yes	506 (28)
no	1284 (72)

The findings presented in Table 4 indicate the demographic differences among the study subjects who were exposed to benzene with or without EKG abnormalities. Of the 1790 subjects, 1083 experienced some type of cardiac function (EKG) abnormality following their exposure to benzene. The remainder of the subjects (N = 707) showed no EKG changes. The demographics, such as male vs. female, adults vs. children, and smoking vs. nonsmoking, were similar between the subjects with and without cardiac abnormalities.

**Table 4.** Table 4. Demographics of the benzene exposed subjects who underwent clinical evaluations in June 2010 – October 2012 – with or without electrocardiogram (EKG) abnormalities

Variable	Participants (N = 1790) [n (%)]	
	with normal EKG (N = 707)	with abnormal EKG (N = 1083)
Gender		
male	411 (58)	633 (58)
female	296 (42)	450 (42)
Age		
<18 years (children)	32 (5)	63 (6)
≥18 years (adults)	675 (95)	1020 (94)
Smoking		
yes	200 (39)	306 (28)
no	507 (61)	777 (72)

The results in Table 5 reveal the changes in the various heart rate parameters in the benzene exposed subjects regardless of their EKG abnormalities. The heart rate parameters included the P wave duration, PR interval, PR segment, QRS duration, QT interval, QTC interval, P wave axis, QRS axis, and T wave axis. Regardless of their EKG abnormalities, a considerable proportion of the benzene exposed subjects experienced notable changes in various heart rate parameters.

**Table 5.** Comparison of various heart rate parameters in benzene exposed subjects who underwent clinical evaluations in June 2010 – October 2012 – with or without electrocardiogram (EKG) abnormalities

Parameter	Participants (N = 1790)			
	with normal EKG (N = 707)		with abnormal EKG (N = 1083)	
	n	n (%)	n	n (%)
P wave duration	512		791	
<80 ms		19 (4)		37 (4)
≥80 ms		493 (96)		760 (96)
PR interval	707		1054	
<200 ms		700 (99)		999 (95)
≥200 ms		7 (1)		55 (5)
QT interval	706		1066	
<440 ms		693 (98)		970 (91)
≥440 ms		13 (2)		96 (9)
QRS interval	706		1065	
<100 ms		619 (88)		860 (81)
≥100 ms		87 (12)		205 (9)
QTC interval	705		1066	
<400 ms		321 (46)		385 (36)
≥400 ms		384 (54)		681 (64)
P wave axis	705		1054	
<+75°		548 (78)		812 (77)
≥+75°		157 (22)		242 (23)
QRS wave axis	705		1065	
<+190°		705 (100)		1065 (100)
≥+190°		0 (0)		0 (0)
T wave axis	706		1065	
<90°		702 (99)		1028 (98)
≥90°		4 (1)		26 (2)

**Figure 2.**
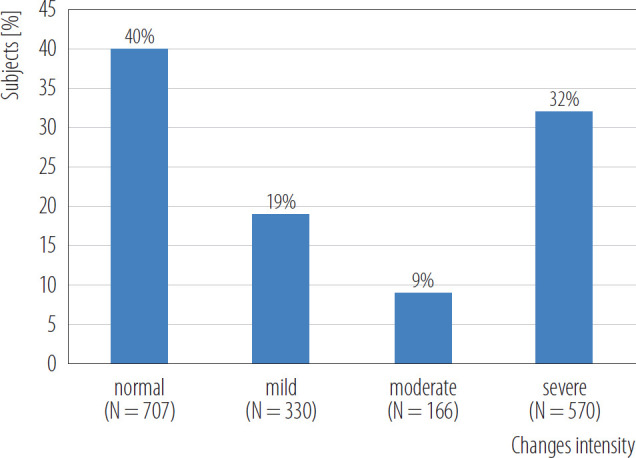
The intensity of changes in the cardiac function among benzene exposed subjects (N = 1790) who underwent clinical evaluations in June 2010 – October 2012.

The intensity of the changes in the cardiac function among benzene exposed subjects is illustrated in Figure 2. The cardiac function abnormalities included ventricular conduction delays, anterior fascicular blocks, sinus rhythm nonspecific T waves, sinus bradycardia ST and T wave abnormalities, sinus rhythm early repolarization, and ventricular hypertrophy. The changes in the cardiac functions were categorized as normal, mild, moderate or severe based on their intensity. Of the 1790 benzene exposed subjects, 1773 were evaluable for cardiac function. Of these 1773 subjects, 707 (40%) had normal cardiac function. Mild abnormal cardiac functions were observed in 330 (19%) benzene exposed subjects. Whereas 166 (9%) benzene exposed subjects had moderate and 570 (32%) had severe cardiac function abnormalities (Figure 2).

The findings in Table 6 reveal the influence of gender and age on the intensity of the changes in the cardiac function among benzene exposed subjects. The intensity of cardiac changes after benzene exposure were pronounced significantly higher in female subjects compared with the male subjects (M±SD 2.28±0.90 vs. 2.14±0.86, p ≤ 0.007). However, no significant changes were found in the intensity of cardiac changes between children (<18 years) and adult (≥18 years) subjects who were exposed to benzene (M±SD 2.18±0.87 vs. 2.22±0.87, p = 0.421). Subgroup analysis indicated that adults aged ≥60 years experienced significantly higher intensity of cardiac changes than children (<18 years) or adults between the age of 18–<40 years or 40–60 years (Table 6).

**Table 6.** Influence of gender and age on the intensity of electrocardiogram (EKG) changes^*^ among benzene exposed subjects who underwent clinical evaluations in June 2010 – October 2012

Variable	Participants (N = 1066) [n]	Intensity of EKG changes* (M±SD)	p
Gender			
male	439	2.14±0.86	
male vs. female	627	2.28±0.90	0.007
Age			
<18 years	58	2.18±0.87	
<18 years vs. ≥18 years	1008	2.22±0.87	0.421
<18 years	58	2.18±0.87	
<18 years vs.18 to <40 years	442	2.13±0.90	0.280
<18 years vs. 40 to <60 years	414	2.25±0.88	0.340
<18 years vs. ≥60 years	152	2.48±0.83	0.016
18 to <40 years	442	2.13±0.90	
18 to <40 years vs. 40 to <60 years	414	2.25±0.88	0.022
18 to <40 years vs. ≥60 years	152	2.48±0.83	0.002
40 to <60 years	414	2.25±0.88	
40 to <60 years vs. ≥60 years	152	2.48±0.83	0.003

* The intensity of the EKG changes computed using the total score derived from categories of mild being representing 1, moderate representing 2, and severe representing 3 as shown in Table 2.

Alterations in the pulmonary function, urinary phenol, and β_2_-microglobulin levels among the benzene exposed subjects regardless of their EKG abnormalities are presented in Table 7. Of the 707 subjects who had normal EKG, 295 (42%) experienced pulmonary function abnormalities following their exposure to benzene. Similarly, a compromised pulmonary function was seen in 548 (51%) of 1083 subjects who had abnormal EKG changes. The levels of urinary excretion of phenol were assessed in the benzene exposed subjects regardless of their EKG abnormalities. Elevated urinary phenol levels were seen in both normal EKG (622 [88%] of the 707) and abnormal EKG (976 [90%] of the 1083) subjects who were exposed to benzene. Abnormal β_2_-microglobulin levels were seen in 59 (8%) of the normal EKG subjects and in 113 (11%) of the abnormal EKG subjects. Statistical analysis indicated that a significantly higher proportion of subjects with abnormal EKGs experienced alterations in the pulmonary function, urinary phenol, and β_2_-microglobulin than those with normal EKGs (p < 0.05) (Table 7).

**Table 7.** Comparison of changes in pulmonary function test (PFT), urinary phenol, and β_2_-microglobulin in benzene exposed subjects who underwent clinical evaluations in June 2010 – October 2012 – with or without electrocardiogram (EKG) abnormalities

Parameter	Participants (N = 1790) [n (%)]		p
	with normal EKG (N = 707)	with abnormal EKG (N = 1083)	
PFT			
normal	412 (58)	535 (49)	0.053
abnormal	295 (42)	548 (51)	0.027
Urinary phenol			
no	85 (12)	107 (10)	0.056
yes	622 (88)	976 (90)	0.038
β_2_-microglobulin			
normal (<2.0 µg)	648 (91)	970 (89)	0.042
abnormal (≥2.0 µg)	59 (8)	113 (11)	0.025

A summary of the findings of the severity of the PFT changes and urinary phenol levels in the benzene exposed subjects regardless of their EKG abnormalities are presented in Table 8. Of the 295 subjects who had normal EKGs but abnormal pulmonary functions, 155 (53%) and 140 (47%) experienced either mild or moderate PFT abnormalities, respectively. No severe PFT changes were observed in this group of benzene-exposed subjects. Of the 548 subjects who had both EKG and PFT abnormal functions, 225 (41%), 235 (43%), and 88 (16%) experienced either mild, moderate, or severe PFT abnormalities, respectively. Of the 622 benzene-exposed subjects who had normal EKG functions, 115 (18%), 192 (31%), 98 (16%), 71 (11%), 90 (15%), and 56 (9%) had either mild-low (<2.5 mg/l), mild-high (<5.0 mg/l), moderatelow (<7.5 mg/l), moderate-high (<10 mg/l), severe-low <20 mg/l), or severe-high 114 (≥20 mg/l) excretion of urinary phenol levels. In the 976 benzene-exposed subjects who had abnormal EKG functions, 200 (20%), 277 (28%), 143 (15%), 100 (10%), 142 (15%), and 114 (12%) had either mild-low (<2.5 mg/l), mild-high (<5.0 mg/l), moderate-low (<7.5 mg/l), moderate-high (<10 mg/l), severe-low <20 mg/l), or severe-high 114 (≥20 mg/l) excretion of urinary phenol levels. Furthermore, the findings show that a significantly higher proportion of subjects with abnormal EKGs, in each subgroup, experienced alterations in the PFTs and urinary phenol when compared to those with normal EKGs (p < 0.05) (Table 8).

**Table 8.** Comparison of the severity of changes in pulmonary function test (PFT) and urinary phenol in benzene exposed subjects who underwent clinical evaluations in June 2010 – October 2012 – with or without electrocardiogram (EKG) abnormalities

Parameter	Participants (N = 1790) [n (%)]		p
	with normal EKG (N = 707)	with abnormal EKG (N = 1083)	
PFT changes			
total	295 (100)	548 (100)	0.027
mild	155 (53)	225 (41)	0.044
moderate	140 (47)	235 (43)	0.033
severe	0 (0)	88 (16)	0.000
Urinary phenol			
total	622 (100)	976 (100)	0.038
mild			
to low (<2.5 mg/l)	115 (18)	200 (20)	0.030
to high (<5.0 mg/l)	192 (31)	277 (28)	0.044
moderate			
to low (<7.5 mg/l)	98 (16)	143 (15)	0.043
to high (<10 mg/l)	71 (11)	100 (10)	0.046
severe			
to low (<20 mg/l)	90 (15)	142 (15)	0.037
to high (≥20 mg/l)	56 (9)	114 (12)	0.021

The authors evaluated the incidence of various abnormal heart rate parameters in the benzene exposed subjects and compared them with those of the general unexposed population reported by various investigators in multiple studies. The findings in Table 9 compare the incidence of the cardiac abnormalities seen in the general unexposed population with those of the authors' study of the benzene exposed subjects. The authors found that the incidence of the EKG abnormalities was 61% in the authors' benzene exposed subjects compared to only 5.2% found in the general unexposed population [32]. Simi larly, the incidence of the P wave, PR interval, QRS interval, QTc, and ST wave interval abnormalities was 96%, 4%, 17%, 60%, and 28% in benzene exposed subjects compared to 16%, 2.1%, 4.5%, 8.7%, and 2.5% in the general population, respectively [32–35].

**Table 9.** Comparison of various heart rate parameters in benzene exposed subjects who underwent clinical evaluations in June 2010 – October 2012 with the general population reported in various studies

Parameter	Participants [%]		Reference
	this study	other studies	
Overall EKG abnormality (N = 1083/1790 evaluable subjects)	61	5.2	De Bacquer et al., 2000 [32]
P wave abnormality (N = 1253/1303 evaluable subjects)	96	16.0	Lehtonen et al., 2017 [35]
PR interval abnormality (N = 62/1761 evaluable subjects)	4	2.1	Holkeri et al., 2020 [33]
QRS interval abnormality (N = 292/1771 evaluable subjects)	17	4.5	Holkeri et al., 2020 [33]
QTC interval abnormality (N = 1065/1771 evaluable subjects)	60	8.7	Montanez et al., 2004 [34]
ST wave abnormality (N = 299/1072 evaluable subjects)	28	2.5	De Bacquer et al., 2000 [32]

EKG – electrocardiogram.

## DISCUSSION

Human exposure to benzene is associated with serious and deleterious health effects; specifically its toxic effects have been seen to affect multiple organ functions including the myocardial, pulmonary, hepatic, renal, nervous, and bone marrow functions [36,37]. These detrimental effects of benzene exposure in individuals have become a major worldwide public health concern. Therefore, a thorough understanding of the health consequences of benzene exposure in humans is important for developing approaches to assess the health risk in those affected individuals. Given its known toxic effects, the large quantity of benzene release that occurred during the BP refinery disaster and the prolonged (40 days) exposure to these toxic chemical emissions by the nearby communities has threatened the health of the residents of the surrounding areas in and near Texas City.

The authors initiated multiple studies to investigate the adverse health effects of the prolonged benzene exposure in those individuals who lived in near Texas City or work by the BP refinery facility [18,28–30,38–42]. This study sought to characterize any adverse changes in cardiac functions that were associated with the benzene exposure from the prolonged toxic BP release. This study, to the best of the authors' knowledge, is the first and largest of its kind to assess the myocardial dysfunction seen in people who were subjected to prolonged benzene exposure. In addition, the authors examined other important physiological parameters including the changes in pulmonary function, urinary excretion of phenol, and serum β_2_-microglobulin levels seen among those subjects exposed to benzene who presented either with or without EKG abnormalities.

The findings of this study reveal that benzene exposure results in measurable EKG changes in those subjects the authors studied. Over 70% of the study subjects experienced some type of abnormality in their cardiac function after their exposure to benzene. These EKG abnormalities included ventricular conduction delays, anterior fascicular blocks, sinus rhythm nonspecific T waves, sinus rhythm early repolarization, and ventricular hypertrophy. Furthermore, female subjects and subjects aged ≥60 years were more vulnerable to these cardiac changes following exposure to benzene. Abplanalp et al. [4] evaluated the cardiovascular effects of benzene in both mice and humans. These authors found that benzene exposure was associated with increased cardiovascular abnormalities.

In this study, the authors examined the combination of parameters, specifically EKG changes that were also associated with PFT, urinary phenol, and β_2_-microglobulin levels among the benzene exposed subjects. It is known that benzene exposure is associated with a deterioration of pulmonary functions in humans. Sharma et al. [43] reported that a decline in the lung functions of petrol pump workers could be due to exposure to petrol fuel vapors, diesel exhaust and airborne particulate matter at petrol pumps. Similarly, a quantitative analysis by Kesavachandran et al. [44] showed reduced lung functions and thereby an increase in respiratory impairment in petrol-pump workers. Other investigators [45–47] also have found adverse effects of petrol/diesel fumes and oil spill exposure on pulmonary functions. In this study, the authors also observed that benzene exposed subjects also experienced pulmonary function abnormalities regardless of their EKG changes.

Urinary phenol determinations have traditionally been used for monitoring benzene exposure [48–50]. Therefore, in this study, the authors evaluated the excretion of phenol in the urine of benzene exposed subjects. In general, clinicians believe that subjects who are not exposed to benzene should excrete undetectable levels of phenol in their urine. Earlier studies have found that the amount of phenol excreted in the urine is well correlated with the amount of the benzene exposure [51,52]. In this study the authors found that >85% of the benzene exposed subjects had considerable amount of phenol in their urine. However, the amount of phenol excretion in the urine of the benzene exposed subjects did not correlate directly with the cardiac abnormalities observed in the study. Moreover, the urinary excretion of phenol indicates that the benzene had circulated in their blood following their exposure. In the authors' earlier studies, the authors observed that benzene exposure had resulted in a considerable amount of urinary phenol excretion in exposed subjects [39,40].

The levels of β_2_-microglobulin in the human serum is considered to be a physiological marker for the activation of the cellular immune system, as well as a tumor marker in certain hematologic and solid malignancies [53]. Therefore, in this study the authors also measured the levels of β_2_-microglobulin in the serum of those benzene exposed subjects with or without EKG abnormalities. A level of ≥2.0 µg of serum β_2_-microglobulin was found in 172 (9.6%) of the 1790 subjects who were exposed to benzene indicating a minimal impact of the benzene exposure on the levels of β_2_-microglobulin in the serum. However, the changes in the serum levels of β_2_-microglobulin in those benzene exposed subjects occurred independently regardless of their EKG changes.

In this study, the authors also examined both the severity of PFT changes and the urinary excretion of phenol levels among the benzene exposed subjects regardless of their EKG changes. The changes in the severity of the subjects' pulmonary functions or their urinary phenol excretion levels were similar between the subjects who had EKG changes or those with no EKG changes after their exposure to benzene. These findings indicate that the changes in the pulmonary functions or the urinary phenol levels were independent of benzene induced EKG changes.

The health effects of benzene exposure that were seen in cardiac and pulmonary functions together with the changes in β_2_-microglobulin or urinary phenol levels may be dependent on the distance from the site of the refinery disaster to the location/address of the study participants who were exposed to the benzene released. Although the authors did not analyze those observed changes in the cardiac and pulmonary functions or changes in the β_2_-microglobulin or urinary phenol levels in this study, the authors' previous studies have demonstrated that most of the observed cardiac abnormalities (irregular heartbeats and heart palpitations) and other health effects of benzene exposure occurred within 5–10 miles distance from the origin of the disaster site [39,40]. However, further studies are required to confirm the relationship between the distance of the subject affected and the epicenter of the source of the benzene release. The findings from this study as well as from the authors' previous studies [18,28–30,38–42] collectively indicate that benzene exposure can affect multiorgan system leading to the impairment of hemopoietic, hepatic, cardiovascular, pulmonary, and urological functions. The toxic effects of benzene could be due to its metabolites that are produced after entering into the body. Benzene enters the body primarily through inhalation of contaminated air or through direct contact with the skin. It is readily absorbed into the body when inhaled into the lungs and excreted as metabolites such as phenol, benzene oxide, benzoquinone, muconaldehydes, hydroquinone, and catechol in the urine [21]. Benzoquinone and muconaldehydes are the most toxic agents among all benzene metabolites. Although it is not known precisely, benzene-induced toxicity appears to involve multiple mechanisms such as oxidative stress, DNA damage, disruption of the cell cycle, and programmed cell death [54–56]. In addition, immune dysfunction may contribute to benzene toxicity due to its interference with innate, humoral and cellular immunity [57,58].

The authors' study has certain limitations. Foremost, this study was conducted using a cross-sectional design. Since clinical outcomes were measured at one time point, this study design has limitations in inferring a causality following benzene exposure. The study findings, however, still allow the generating of a hypothesis for further investigation. Another limitation of this study was the lack of baseline data prior to the flaring event at the BP refinery. Since such disasters are not planned events, it is impossible to have had any baseline data for these subjects prior to their exposure to the uncontrolled toxic emissions. Subjects' comorbidities and their lifestyle, may have influenced the observed findings. In addition, this investigation was retrospective in nature. Nonetheless, the findings of the authors' study indicate that the benzene exposure from the refinery disaster is associated with abnormal cardiac and pulmonary functions among the exposed subjects.

## CONCLUSIONS

In conclusion, the findings of this study indicate that the benzene exposure that resulted from the BP flaring incident adversely affected the cardiac functions in those individuals who were exposed to it. In addition, those benzene exposed subjects also experienced abnormal pulmonary functions regardless of their EKG changes. In the authors' previous studies, the authors have found that benzene exposed subjects experienced detrimental health effects including impairment of the hematological, hepatic, renal, and other organ functions after the BP flaring disaster. Together, these findings demonstrate that the physiological impairment occurs in multiple organs including the myocardium, pulmonary, hematological, hepatic, and renal functions represent those individuals who have been exposed to significant amounts of toxic benzene. In addition, those individuals who experience more advanced deterioration of their organ functions may have been exposed to higher dose and are thus more sensitive to these toxic chemical compounds and prone to a higher risk of developing a myriad of malignancies in the future.
